# Confounding influences of malnutrition and *Plasmodium falciparum* and *Schistosoma haematobium* infections on haematological parameters in school children in Muyuka, Cameroon

**DOI:** 10.1186/s12879-021-06201-9

**Published:** 2021-05-25

**Authors:** Irene Ule Ngole Sumbele, Ofon Vitalis Otia, Lorraine Francis, Orelien Sylvain Mtopi Bopda, Calvin Bisong Ebai, Teh Rene Ning, Helen Kuo Kuo Kimbi, Theresa Nkuo-Akenji

**Affiliations:** 1 Department of Zoology and Animal Physiology, University of Buea, Buea, Cameroon; 2 Department of Microbiology and Immunology, College of Veterinary Medicine, Cornell University, Ithaca, New York USA; 3 Department of Population Medicine and Diagnostic Services, College of Veterinary Medicine, Cornell University, Ithaca, New York USA; 4 Department of Medical Laboratory Sciences, Faculty of Health Sciences, University of Buea, Buea, Cameroon; 5 Department of Medical Laboratory Science, Faculty of Health Sciences, University of Bamenda, Bambili, Cameroon; 6 Department of Microbiology and Parasitology, University of Buea, Buea, Cameroon

**Keywords:** Anaemia, Co-infection, Haematological parameter, Malnutrition, *Plasmodium*, School-aged children, Stunting, *S. Haematobium*, Cameroon

## Abstract

**Background:**

School-aged children (SAC) are a high-risk demographic group for infectious diseases and malnutrition. The objective of this study was to assess the burden and the effect of *Plasmodium falciparum* and *Schistosoma haematobium* infections on the haematological indices in SAC and the confounding influence of malnutrition on the outcomes.

**Methods:**

This cross-sectional study was conducted in SAC 4–14 years old living in Ikata, Bafia and Mile 14-Likoko in Muyuka, Cameroon. Anthropometric measures of malnutrition were obtained and blood samples collected were used for detection of malaria parasites by Giemsa-stained blood films using light microscopy and complete blood count analysis using an automated haematology analyser. Urine samples collected were used to detect micro haematuria with the aid of reagent strips and the eggs of *S. haematobium* by urine filtration technique. Multiple linear regression model was used to examine influence of independent variables on haematological parameters.

**Results:**

Out of the 606 SAC examined, the prevalence of single infections with *Plasmodium* or *S. haematobium* and co-infection with both parasites was 16.2, 16.3 and 8.3%, respectively. Overall, malaria parasite (MP), urogenital schistosomiasis, malnutrition, anaemia, haematuria, microcytosis and thrombocytopenia was prevalent in 24.4, 24.6, 25.9, 74.4, 12.2, 45.4 and 11.1% of SAC, respectively. A significant linear decline (*P* = 0.023) in prevalence of *P. falciparum* infection with the severity of stunting was observed. Factors that significantly influenced haematological parameters included haemoglobin: age, stunting and MP; haematocrit: age and MP; white blood cell count: age; red blood cell count; age and MP; lymphocyte counts: stunting; mean cell volume: age; mean cell haemoglobin: age and stunting; mean cell haemoglobin concentration: sex, stunting and red cell distribution width-coefficient of variation: sex, age and stunting.

**Conclusions:**

Malnutrition, *Plasmodium* and *S. haematobium* infections are common while anaemia is a severe public health problem in Muyuka, Cameroon. The interaction between haematological parameters with malaria parasites as well as linear growth index was negative and other interactions indicate systemic inflammation. While findings provide contextual intervention targets to ensure the judicious use of the limited resources, there is need for regular monitoring and proper treatment to improve the health of the underserved population.

**Supplementary Information:**

The online version contains supplementary material available at 10.1186/s12879-021-06201-9.

## Background

Malaria caused by protozoan parasites such as *Plasmodium falciparum* and urogenital schistosomiasis (UGS) caused by the trematode helminth *Schistosoma haematobium* impose tremendous public health burdens in tropical and subtropical countries. Both diseases have been associated with poverty and factors such as low socio-economic status, poor sanitation, limited access to safe water, poor education and poor awareness, which also play a key role in their transmission [1–3].

Beyond the pre-school years, school-aged children (SAC) are a high-risk demographic group for infectious diseases and malnutrition. Malaria whose symptoms and signs (anaemia, fever, headaches, vomiting, nausea, abdominal pain, inappetence, bitter mouth, dizziness, and weakness) may be more subtle in partially immune children, is increasingly an important challenge in SAC even though they have attracted relatively little attention as a group in need of special protection measures [4, 5]. Previous studies have underappreciated the burden amongst whom the prevalence of infection is habitually higher than that among children less than 5 years old and adults [6–8]. Following the scale-up of treated bed nets across the country between 2000 and 2015, a significant decrease in the overall prevalence of malaria cases from 41 to 24.3% was reported in Cameroon [9]. With the changing dynamics of malaria transmission and infection due to interventions, which include case management using artemisinin-based combination therapy (ACT) drugs and vector control through the large-scale distribution of long-lasting impregnated net (LLINs), monitoring the changes in morbidity in this age group is invaluable.

Like most neglected tropical diseases (NTD), schistosomiasis is a chronic and debilitating illness with the ability to affect child development and productivity. Children aged 5–17 years in developing countries are at highest risk of infection and are the most infected group [10, 11]. Schistosomiasis is likely to cause anaemia, stunting and a reduced ability to learn although the effects are usually reversible with treatment [12]. In Cameroon, rural areas are the most affected with UGS. However recent report highlights the emergence of urban urogenital schistosomiasis in the Mount Cameroon area in Tiko Health District, which can be attributed to the migration of individuals from the conflict hit areas of Kumba, Munyenge and Kotto-Barombi [13]. The prevalence of *S. haematobium* in the Mount Cameroon area ranges from 25.4–40.27% although, the annual mass drug administration (MDA) campaigns control strategy in SAC has considerably reduced the egg-patent prevalence of the disease [14–17]. However, after several rounds of localized MDA campaigns and the fact that transmission dynamics and re-infection patterns post-treatment are complex [18], monitoring the variation of intensity of infection and associated morbidity is crucial in ascertaining the sustained impact of control measures or react to new outbreaks.

Since SAC are often under-represented in infectious diseases community-based cluster surveys, malaria and UGS burden in this group is poorly defined. Hence, the objective of this study was to assess the burden and the influence of infections with *P. falciparum* and *S. haematobium* on haematological parameters in SAC and the confounding influence of malnutrition on the outcomes in order to provide an insight on morbidities associated with co-infections in areas with ongoing interventions in place. The findings will provide contextual intervention targets in the community to ensure the judicious use of the limited resources in improving the health of the underserved population.

## Methods

### Study area and participants

The study was carried out in the schistosomiasis endemic foci of Ikata, Bafia and Mile 14-Likoko, which are three rural localities in the Muyuka Health District. The study sites have been described in detailed by Ebai et al. [19]. Environmental and socio-economic conditions in these areas favour the thriving of the vectors and the transmission of these parasites. A prevalence of malaria parasite (MP) of 35.5% and UGS of 34.3% was reported in the area in a study of a cross section of the population [7, 14]. Intervention measures in the area include but are not limited to the free distribution of LLINs to pregnant women and children and the mass distribution of mebendazole by the Ministry of Public Health in Cameroon to SAC in schools.

This study was conducted among SAC aged 4-14 years of both sexes whose parents consented to their participation in the study. As an inclusion criterion, only children who had resided for at least 3 months in the study area were enrolled in the study and their participation in the study was voluntary.

### Study design, sample size estimation and sampling

This cross-sectional study was carried out between March to June 2015 to coincide with the malaria and schistosomiasis transmission season. This was a repeated cross-sectional study following intervention studies in the previous transmission season [7, 14]. The sample size of participants to be examined was determined using the formula n = Z^2^pq/d^2^ [20], where n was the sample size required; Z was 1.96, which is the standard normal deviate (for a 95% confidence interval, CI); p was 35.3% or 34.3%, the proportion of malaria parasite or UGS prevalence reported previously in the area [7, 14]; q was 1-p, the proportion of MP or UGS negative; and d was 0.05, the acceptable error willing to be committed. The optimum sample size was estimated to be 349 (359.5 + 346.3/2). To mitigate against possible loss of samples due to blood clotting and withdrawal from the study, the sample size was increased by 15% for a minimum of 401 SAC. With respect to sampling to obtain a representative minimum sample of 401 firstly, 8 primary schools from a list of schools from the three study sites of Likoko, Ikata and Bafia were selected randomly. Secondly, participants were selected at random by balloting from each class accounting for the numbers above the calculated sample size. Likoko village with the highest number of primary schools had the highest number of study participants followed by Ikata and Bafia.

### Implementation of study

The inhabitants of Likoko, Ikata and Bafia were educated on the importance, benefits and protocol of the study in several reconnaissance visits made to the localities prior to the commencement of the study. Children who presented consent forms signed by parent / caregiver were enrolled into the study and information on both demography and factors that may be associated with malaria and UGS were obtained through an interview using a simple structured questionnaire. Clinical evaluation by the study nurse was carried out subsequently where weight, height and temperature were measured. The study involved the collection of venous blood and one urine sample for haematological analysis, and microscopic detection of *S. haematobium* eggs, respectively. Labelled blood and urine samples placed on ice blocks were transported to the University of Buea Malaria Research Laboratory for further analysis.

### Questionnaire administration and clinical evaluation

A pre-tested questionnaire was administered to each participant with the aid of the teachers to obtain information on demography, hygienic practices, possible risk factors of *Plasmodium* and helminth infections as well as malnutrition and anaemia. The ages of participants were obtained from the school register.

The axillary temperature was measured using a digital thermometer and a participant with body temperature ≥ 37.5 °C was considered febrile.

Height was measured to the nearest 0.1 cm using a graduated ruler of length 2 m. Body mass was measured to the nearest 0.5 Kg using a mechanical scale of capacity 120 Kg (KINLEE® model BR9310), and upper arm circumference was measured to the nearest 0.1 cm using a graduated tape. These measurements were used to calculate an array of anthropometric indices used as proxies for malnutrition: weight-for-age (WA: under-weight); height-for-age (HA: stunting); weight-for height (WH: wasting). Anthropometric indices were computed as z-scores based on the WHO growth reference curves using the WHO AnthroPlus for personal computers manual [21]. A child was identified as being malnourished if he or she scored < − 2 in one of the anthropometric indices. A z-score between < − 2 and ≤ − 3 was considered as moderate wasting, moderate stunting or moderate underweight while Z scores of < − 3 indicated severe wasting, severe stunting or severe underweight [22].

### Malaria parasite diagnosis and full blood count

From each participant, approximately 2 ml of venous blood was collected in ethylenediamine tetra-acetate tubes for malaria parasite detection and haematological analysis. Thick and thin blood films were prepared in situ. The thin blood films were fixed in absolute methanol and together with the thick blood films were Giemsa stained and examined microscopically following standard procedures [23]. Slides were considered positive when asexual forms and/or gametocytes of any *Plasmodium* species were observed on the blood film. All the slides were read twice by two independent microscopists. Malaria parasite per μL of blood was established by counting the number of parasites per 200 leukocytes and multiplying by the persons white blood cell (WBC) count. Parasitaemia was classified as low (≤ 500 parasite /μL of blood), moderate (501–5000 parasites/μL of blood) and high (> 5000 parasites/μL of blood) [24].

A complete blood count analysis was done using a Beckman coulter counter (URIT 3300) which automatically gave values for red blood cell (RBC), WBC and platelet counts, haemoglobin (Hb), haematocrit (Hct), mean cell volume (MCV), mean cell haemoglobin (MCH), mean cell haemoglobin concentration (MCHC) and red cell distribution width-coefficient of variation (RDW-CV) following the manufacturer’s instructions. The classification of anaemia (Hb concentration below the WHO reference values for age or gender) and its severity was done in accordance with WHO standards (mild anaemia = 10–10.9 g/dL, moderate anaemia = 7–9.9 g/dL and severe anaemia < 7 g/dL) [23, 25].

### Urine analysis for haematuria and schistosome eggs

Each study participant collected approximately 25 mL of midstream urine into a screw cap vials after a brisk exercise between 10 am and 2 pm. Gross haematuria was determined by visual observation while micro haematuria was determined with the aid of reagent strips (combistix) following the manufacturer’s guide (CYBOW™ 11 M a series of Health Mate Ref 0974). Eggs of *S. haematobium* were detected using the urine filtration technique. Following agitation, 10 mL of urine was drawn using a syringe and filtered through a polycarbonate membrane filter (STERLITECH corporation, USA). The filter membrane was examined microscopically for the presence of *S. haematobium* eggs as described by Cheesbrough [23]. *S. haematobium* egg density was expressed as the number of eggs in 10 mL urine (e/10 mL) and the intensity of infection was categorised as either light (< 50 e/10 mL) or heavy infection (≥ 50 e/10 mL) [26, 27].

### Data analysis

Descriptive measures such as the mean and standard deviation (SD), geometric means, frequencies, and proportions were used to summarize data. Differences in proportions between populations were compared using Chi (χ^2^) test. The attributable risk (AR%) of anaemia caused by malaria, UGS and stunting was calculated accordingly [28]: [(n_*1*_*m*_*0*_ − *n*_*0*_*m*_*1*_)/*n*(*n*_*0*_ *+ m*_*0*_)] × 100, where n_0_ = anaemic children without malaria/UGS/stunting and n_1_ = anaemic children with malaria/UGS/stunting, whereby n_0_ + n_1_ = n, m_0_ = non anaemic children without malaria/UGS/stunting, and m_1_ = non anaemic children with malaria/UGS/stunting, whereby m_0_ + m_1_ = m. Geometric means were computed for those positive only and the log transformed counts were used in the analysis. Geometric mean parasite density (GMPD) of *P. falciparum* and geometric mean egg count (GMEC) of *S. haematobium* by age, sex and nutritional status and severity were compared by the Student’s t-test, and Mann Whitney U test where appropriate and the mean haematological parameters were compared by analysis of variance (ANOVA). Potential confounders of haematological values to be entered into a multiple linear regression (MLR) model were identified after exploratory analysis. Any potential confounder with a moderate (*P* < 0.2) relation with both the dependent variable and the confounder of interest was included in the later MLR models. The 95% confidence interval (CI) was reported and *P*-values < 0.05 were considered suggestive of statistical significance. All data was analysed using IBM-Statistical Package for Social Science (SPSS) version 21 (IBM-SPSS Inc., Chicago, IL, USA).

### Ethical considerations

The study protocol was reviewed and approved by the Institutional Ethical Review Board hosted by the Faculty of Health Sciences, University of Buea following administrative authorisation from the Regional Delegation of Public Health and Basic Education. The ethical approval reference for the study is 2014/243/UB/FHS/IRB. The study was conducted in accordance with the World Medical Association (WMA) principles as stated in the Declaration of Helsinki. The population was sensitized in their various communities at the beginning of the study. Written informed consent was obtained from all parents/caregivers whose child/children participated in the study after explaining the purpose and benefits of their participation. Participation was totally voluntary, and a participant could decide to halt their participation in the study at any time without any penalty. Participants who had malaria and or urogenital schistosomiasis were given first line treatment as recommended by the national treatment guideline policy for uncomplicated malaria and helminths.

## Results

### Characteristics of participants

The characteristics of the 606 SAC examined is presented in Table 1. The mean (SD) age of the study participant was 8.94 (2.1) years with no significant difference between sex and age. The majority (59.2%) of the SAC were enrolled from the Likoko locality. The clinical profile of the participants revealed a prevalence of anaemia, malaria parasite, UGS, haematuria, microcytosis and thrombocytopenia of 74.4, 24.4, 24.6, 12.2, 45.4 and 11.1%, respectively. While no significant difference in prevalence of malaria parasite was observed with sex and age, significantly higher prevalence of anaemia was observed in children 4–9 years old (78.5%), UGS and haematuria in females (28.6, 14.9%) and thrombocytopenia in those 4–9 years old (13.4%) than their respective contemporaries. The prevalence of microcytosis was significantly higher in males (50.7%) and children 4–9 years (51.7%). Likewise, the prevalence of malnutrition (25.9%) and its forms such as underweight (6.6%) and stunting (22.9%) varied significantly with sex and age with a higher prevalence observed in males and children 10–14 years as shown in Table 1.

**Table 1:** Characteristics of study participants by sex and age

Parameter		Sex			Age group in years		Overall	*P*- value
		Female	Male	*P*-value	4–9	10–14		
% (N)		50.8 (308)	49.2 (298)		59.1 (358)	40.9 (248)	100 (606)	
Mean age (SD) in years		8.97 (2.0)	8.91 (2.3)	0.724	7.50 (1.4)	11.0 (1.1)	8.94 (2.1)	**< 0.001**
Mean height (SD) in cm		126.6 (12.3)	123.7 (12.3)	**0.004**	118.7 (10.1)	134.5 (9.0)	125.2 (12.4)	**< 0.001**
Mean weight (SD) in kg		28.2 (7.0)	27.6 (9.3)	0.431	24.4 (4.7)	33.1 (9.3)	27.9 (8.2)	**< 0.001**
Site % (n)	Bafia	18.2 (56)	14.4 (43)		14.2 (51)	19.4 (45)	16.3 (99)	
	Ikata	25.3 (78)	23.5 (70)	0.315	24.3 (87)	24.6 (61)	24.4 (148)	0.216
	Likoko	56.5 (174)	62.1 (185)		61.5 (220)	56.0 (139)	59.2 (359)	
			Clinical profile					
Fever prevalence (n)		20.1 (61)	23.6 (69)	0.292	22.1 (77)	21.4 (53)	21.8 (130)	0.826
Anaemia prevalence (n)		74.0 (228)	74.8 (223)	0.820	78.5 (281)	68.5 (170)	74.4 (451)	**0.006**
Malaria parasite prevalence (n)		25.6 (79)	23.2 (69)	0.475	24.9 (89)	23.8 (59)	24.4 (148)	0.73
Asymptomatic malaria prevalence (n)		20.8 (64)	17.8 (53)	0.351	19.8 (71)	18.5 (46)	19.3 (117)	0.694
UGS prevalence (n)		28.6 (88)	20.5 (61)	**0.021**	23.5 (84)	26.2 (65)	24.6 (149)	0.440
Haematuria prevalence (n)		14.9 (46)	9.4 (28)	**0.037**	11.2 (40)	13.7 (34)	12.2 (74)	0.348
^a^Underweight prevalence (n)		4.3 (10)	8.9 (20)	**0.044**	5.3 (19)	11.0 (11)	6.6 (30)	**0.042**
Stunting prevalence (n)		16.9 (52)	29.2 (87)	**< 0.001**	17.9 (64)	30.2 (75)	22.9 (139)	**< 0.001**
^b^Wasting prevalence (n)		9.1 (1)	4.5 (1)	0.606	6.1 (2)		6.1 (2)	
Malnutrition prevalence (n)		19.2 (59)	32.9 (98)	**< 0.001**	21.2 (76)	32.7 (81)	25.9 (157)	**0.002**
Leucopenia prevalence (n)		1.9 (6)	2.0 (6)	0.954	2.0 (7)	2.0 (5)	2.0 (12)	0.958
Microcytosis prevalence (n)		40.3 (124)	50.7 (298)	**0.010**	51.7 (185)	36.3 (90)	45.4 (275)	**< 0.001**
Thrombocytopaenia prevalence (n)		11.0 (34)	11.1 (33)	0.989	13.4 (48)	7.7 (19)	11.1 (67)	**0.027**

^a^Underweight was evaluated for 458 SAC

^b^Wasting was evaluated for 33 SAC

*P*-values in bold are statistically significant.

### Prevalence of single and co-infection

The prevalence of single infection with *P. falciparum* or *S. haematobium* and co-infection with both parasites was 16.2, 16.3 and 8.3%, respectively, with no significant variation with sex and age. Significantly higher (*P* < 0.001; *P* < 0.001 and *P* = 0.001) prevalence of single infection with *S. haematobium* (25.9%) and co-infection with *P. falciparum* and *S. haematobium* (13.4%) were observed in SAC from the Likoko locality while single *P. falciparum* infection was observed in Ikata (20.9%), respectively. The higher prevalence observed in the anaemics was not statistically significant when compared with their counterparts as shown in Table 2. The prevalence of *P. falciparum* and *S. haematobium* was lower in children who presented with fever contrary to the significantly higher (*P* = 0.008) prevalence of co-infection observed in those with fever (13.8%). With regard to malnutrition and its forms, the prevalence of *S. haematobium* and co-infection was comparable among the different groups unlike that of *P. falciparum*. The prevalence of *P. falciparum* was significantly lower (*P* = 0.018, *P* = 0.006) in those malnourished (10.2%) or the stunted (8.6%) than those normal, respectively (Table 2).

**Table 2:** Prevalence of single infection with *P. falciparum or S. haematobium* and co-infections by demographic and clinical status

Parameter	Category	N	Single *P. falciparum* infection		Single *S. haematobium* infection		Co-infection	
			Prevalence (n)	*P*-value	Prevalence (n)	*P*- value	Prevalence (n)	*P*-value
All		606	16.2 (98)		16.3 (99)		8.3 (50)	
Sex	Female	308	15.3 (47)	0.535	18.2 (56)	0.212	10.4 (32)	0.052
	Male	298	17.1 (51)		14.4 (43)		6.0 (18)	
Age in years	4–9	358	16.8 (60)	0.637	15.4 (55)	0.436	8.1 (29)	0.872
	10–14	248	15.3 (38)		17.7 (44)		8.5 (21)	
Site	Bafia	99	4.0 (4)		3.0 (3)		0.0 (0)	
	Ikata	148	20.9 (31)	**0.001**	2.0 (3)	**< 0.001**	1.4 92)	**< 0.001**
	Likoko	359	17.5 (63)		25.9 (93)		13.4 (48)	
Anaemia status	Anaemic	451	17.3 (78)	0.200	17.7 (80)	0.111	9.1 (41)	0.200
	Normal	155	12.9 (20)		12.3 (19)		5.8 (9)	
Fever status	Fever	130	10.8 (14)	0.054	15.4 (20)	0.756	13.8 (18)	**0.008**
	Normal	466	17.8 (83)		16.5 (77)		6.7 (31)	
Stunting	Stunted	139	8.6 (12)	**0.006**	16.5 (23)	0.939	7.2 (10)	0.606
	Normal	467	18.4 (86)		16.3 (76)		8.6 (40)	
Underweight	Underweight	30	20.0 (6)	0.731	16.7 (5)	0.937	6.7 (2)	0.835
	Normal	428	17.5 (75)		16.1 (69)		7.7 (33)	
Malnutrition	Malnourished	157	10.2 (16)	**0.018**	17.2 (27)	0.735	7.0 (11)	0.510
	Normal	449	18.3 (82)		16.0 (72)		8.7 (39)	

*P*- values in bold are statistically significant.

**Fig. 1:**
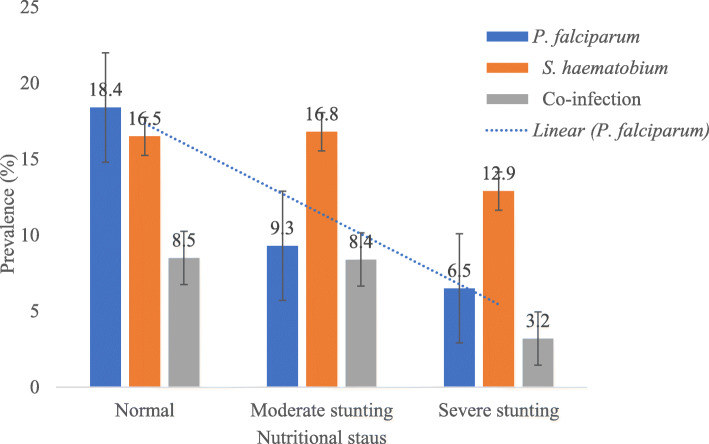
Prevalence of single infections of *P. falciparum* and *S. haematobium* and co-infection by stunting severity

Observation from the study demonstrated a significant linear decline (χ^2^ = 7.516, *P* = 0.023) in prevalence of *P. falciparum* infection with the severity of stunting with those normal having the highest prevalence (18.4%) while those with severe stunting had the least (6.5%). No significant trend in the prevalence of *S. haematobium* and co-infection was observed with the severity of stunting even though the prevalence was lowest in those with severe stunting (12.9 and 3.2%, respectively) as revealed in Fig. 1.

### Malnutrition severity and infection intensity

**Fig. 2:**
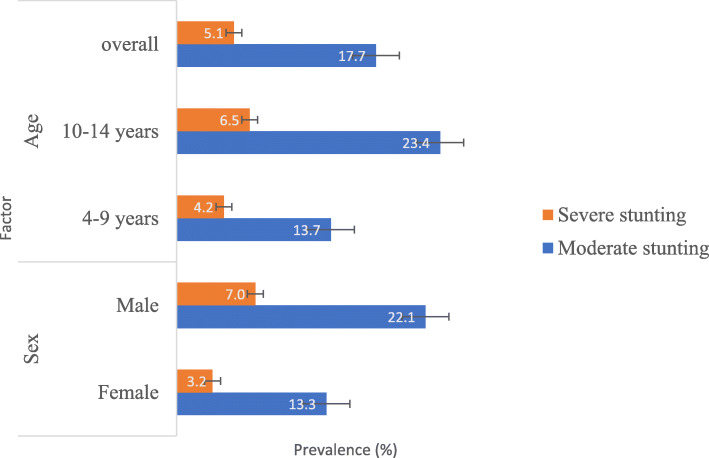
Moderate and severe stunting prevalence by sex and age

The prevalence of moderate and severe stunting was 17.7 and 5.1%, respectively. Males and children 10–14 years old had significantly higher (χ^2^ = 14.105, *P* = 0.001; χ^2^ = 11.986, *P* = 0.002) prevalence of moderate and severe stunting, respectively, as shown in Fig. 2.

*Plasmodium falciparum* parasitaemia ranged from 71 to 33,250 parasites /μL of blood while *S. haematobium* eggs /10 mL of urine ranged from 1 to 494. Although males, children 4–9 years, the malnourished and those with severe stunting had a higher *P. falciparum* GMPD/ μL of blood the differences were not statistically significant. A similar pattern in the distribution of *S. haematobium* GMEC was observed except for sex with females having a higher GMEC (26 eggs/10 mL of urine) than males (20 eggs/10 mL of urine) as shown in Table 3.

**Table 3:** *P. falciparum* GMPD and *S. haematobium* GMEC by sex, age nutritional status and stunting severity

Parameter	Characteristics	*P. falciparum* /μL of blood			*S. haematobium* /10 mL of urine		
		GMPD (n)	Range	*P* value	GMEC (n)	Range	*P* value
Overall		643 (148)	71–33,250		24 (50)	1–494	
Sex	Female	564 (79)	71–12,721	0.156	26 (32)	1–494	0.284*
	Male	747 (69)	140–33,250		20 (18)	1–280	
Age in years	4–9	726 (89)	110–33,250	0.129	25 (29)	1–494	0.472*
	10–14	535 (59)	71–20,600		22 (21)	2–250	
Nutritional status	Normal	642 (126)	71–33,250	0.785*	23 (40)	1–494	0.701*
	Malnourished	675 (27)	168–11,700		29 (11)	1–250	
Stunting Severity	Moderate	641 (19)	168–4230	0.651*	24 (9)	1–250	0.220*
	Severe	674 (3)	270–3603		48 (1)	48	

*P* values obtained by t-test.

*P value obtained by Mann Whitney U-test

**Fig. 3:**
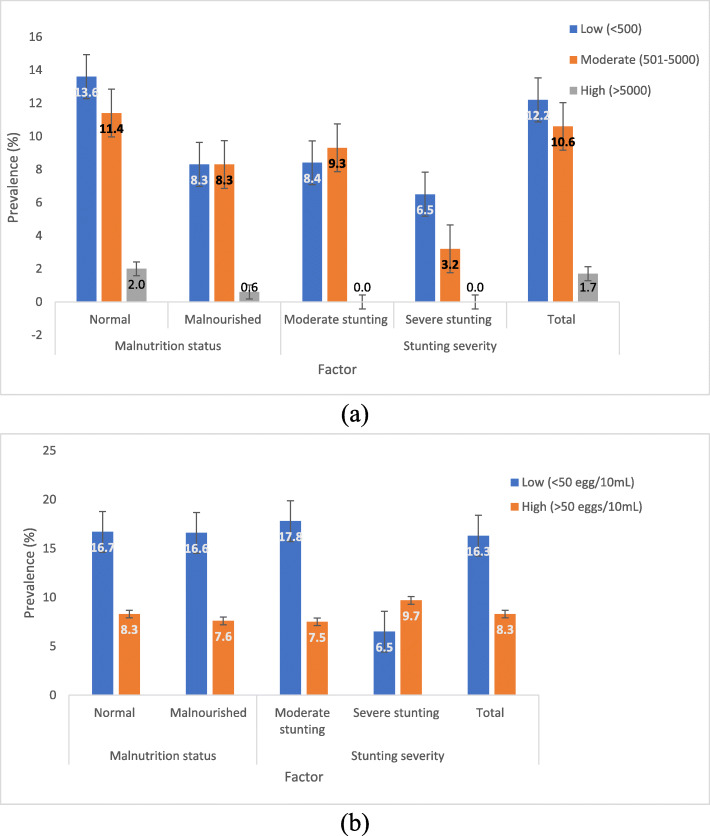
*P. falciparum* (a) and *S. haematobium* (b) parasite density category prevalence by malnutrition status and severity of stunting

Of the 148 SAC infected with *P. falciparum,* the majority (*n* = 74, 50%) had low parasite density. As shown in Fig. 3 (a), the prevalence of low, moderate and high *P. falciparum* density was 12.2, 10.6 and 1.7%, respectively and this was lower in malnourished than well-nourished children. In addition, the prevalence of low and moderate *P. falciparum* density decreased with the severity of stunting. With respect to *S. haematobium*, the prevalence of low and high egg density was respectively, 16.3 and 8.3% and, a comparison with the nutritional status showed no significant differences with the severity of stunting (Fig. 3 (b)).

### Effect on haematological parameters

**Fig. 4:**
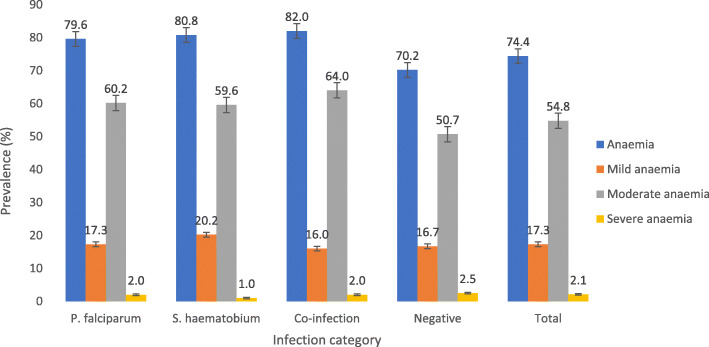
Prevalence of anaemia and its severity by infection category

Anaemia was common in the study population (74.4%) even in SAC negative for both infections (70.2%). The prevalence of anaemia was significantly higher (χ^2^ = 8.375, *P* = 0.039) in children with co-infection (82.0%) when compared with those with single infection and those negative for both infections. Mild, moderate and severe anaemia was prevalent in 17.3, 54.8 and 2.1% of the population, respectively. Although no significant difference (χ^2^ = 10.765, *P* = 0.292) in the severity of anaemia was observed, moderate anaemia was most common in children with co-infection (64%) when compared with their counterparts as shown in Fig. 4. The population attributable risk of anaemia due to the malaria parasite, urogenital schistosomiasis, co-infection with both parasites and stunting in the study population was 2.6, 3.0, 0.9 and 2.8%, respectively.

The mean Hct, WBC, lymphocyte and platelet counts, MCV, MCH, MCHC and RDW-CV were comparable amongst those infected with *P. falciparum*, *S. haematobium*, and co-infection and those negative. Post hoc comparisons revealed a significantly lower (*P* = 0.028, *P* = 0.035) mean Hb (10.5 (1.2) g/dL) concentration and mean RBC (4.1 (4.4) × 10^12^/L) counts in children infected with *P. falciparum* when compared with those negative, respectively (Table 4).

**Table 4:** Mean (SD) haematological parameter as affected by infection category

Parameter	*P. falciparum*	*S. haematobium*	Co-infection	Negative	Overall	*P*-value
N	98	99	50	359	606	
Hb in g/dL	10.5 (1.2)^**a**^	10.5 (1.4)	10.5 (1.3)	10.8 (1.4)^**b**^	10.7 (1.4)	0.059
Hct in %	30.0 (3.5)	30.3 (4.0)	30.2 (3.7)	30.9 (3.9)	30.6 (3.9)	0.157
WBC × 10 ^9^/L	9.5 (4.9)	9.3 (2.7)	9.3 (3.0)	9.9 (5.6)	9.6 (5.0)	0.621
RBC × 10^12^/L	4.1 (4.4)^**a**^	4.2 (5.0)	4.1 (4.7)	4.2 (5.2)^**b**^	4.2 (5.0)	0.135
Lymphocyte x 10^9^L	4.2 (3.5)	3.8 (1.7)	3.9 (2.1)	4.3 (2.8)	4.2 (2.7)	0.451
MCV in fL	73.4 (6.0)	73.2 (6.7)	73.1 (6.1)	73.4 (6.1)	73.3 (6.2)	0.984
MCH in pg	25.5 (2.2)	25.3 (2.3)	25.4 (2.4)	25.5 (2.2)	25.5 (2.2)	0.808
MCHC in g/L	34.8 (1.8)	34.7 (1.8)	34.8 (2.4)	35.0 (2.1)	34.9 (2.0)	0.724
Platelet x 10^9^L	276.2 (111.9)	279.0 (975.0)	253.5 (118.7)	280.9 (113.7)	277.6 (111.3)	0.442
RDW-CV%	12.5 (1.3)	12.7 (1.4)	12.4 (1.2)	12.5 (1.4)	12.5 (1.3)	0.514

^a, b^Means with dissimilar superscript are significantly different

*P* values obtained by ANOVA

A multiple linear regression analysis involving the 606 participants, with the different haematological variables as the dependent variable and sex, age, HAZ (stunting index), MP status, UGS status and co infection as independent variables, revealed that co-infection did not significantly influence any haematological parameter (Additional file 1). Factors of significant influence on haematological parameters included Hb: age (*P* < 0.001), stunting (*P* = 0.004) and MP status (*P* = 0.013); Hct: age (*P* < 0.001) and MP status (*P* = 0.038); WBC: age (*P* = 0.034); RBC: age (*P* = 0.001) and MP status (*P* = 0.034); lymphocyte counts: stunting (*P* = 0.015); MCV: age (*P* < 0.001); MCH: age (*P* < 0.001) and stunting (*P* = 0.001); MCHC: sex (*P* = 0.046) and stunting (*P* = 0.004) and RDW-CV: sex (*P* < 0.001), age (*P* = 0.001) and stunting (*P* = 0.023) as shown in Additional file 1.

## Discussion

Studies in the Mount Cameroon area have demonstrated the significant contributions of co-infection of *Plasmodium* and *S. haematobium* to the burden of anaemia in pregnant women [29] and the persistent transmission of schistosomiasis despite MDA and the burden of schistosomiasis in SAC [15]. In the present study we assessed the burden and influence of these infections on haematological indices in SAC and the confounding influence of malnutrition on the outcomes to provide some insight on the morbidities associated with co-infections in areas of ongoing intervention strategies.

Findings from the study revealed similar prevalence of both *Plasmodium* and *S. haematobium* with most infections being single, light and asymptomatic. A drop in prevalence was observed when compared with findings of studies carried out in the general population in the same area a year earlier [7, 14]. However, in schistosomiasis endemic areas, the outcome of infection with *Plasmodium* is of growing concern. The prevalence of *Plasmodium* (24.4%) is lower than the 33.9% observed in a simultaneous study in SAC in non-schistosomiasis endemic area within the same Muyuka Health District [30]. These findings are, however, comparable with a 26.8 and 27.4% prevalence observed in similar populations in schistosomiasis endemic areas in the West Region of Cameroon [31] and North-western Tanzania, respectively [32]. While the decrease in the prevalence of infection in this cohort of children may be attributed to LLINs use and its community benefit, although SAC have a propensity to use LLINs less frequently, its use remains an effective method of malaria control [4, 7]. Nonetheless, the interactions in epidemiology in schistosomiasis-endemic foci resulting in lower prevalence of *Plasmodium* infection when compared with non-endemic foci warrants further investigation.

Observations from the study revealed no significant differences in the prevalence of both *Plasmodium* and *S. haematobium* with age. On the other hand, UGS was more common in females with haematuria being a common manifestation consistent with other studies [33, 34]. This higher prevalence in females is consistent with observations made by Noriodea et al. [35] in Nigeria but contrasts those of Mewabo et al. [34] and Njunda et al. [36] in Cameroon and Abdulkareem et al. [37] in Nigeria. Although a higher prevalence of UGS in females may be attributed to their higher frequency of contact with infested water, for peri domestic activities, it is worth noting that limited access to safe water and sanitation conditions are implicated in the transmission of both *Plasmodium* and *S. haematobium* infections [2, 3].

The prevalence of *Plasmodium* and *S. haematobium* co-infection in SAC (8.3%) is like that reported in Gabon [38], higher in Ghana [39] and lower when compared with some areas in Nigeria [40]. Ikata, Bafia and Mile 14-Likoko in Muyuka, Cameroon are *S. haematobium* endemic areas due to the presence of an efficient *Bulinus* snail vector and the use of untreated waterways for domestic, farming and recreational use. Risk of co- infection with *Plasmodium* in these areas is also high as these areas are part of the holoendemic stratum with high and perennial malaria parasite transmission [41]. Furthermore, environmental variants within the different localities maybe of significance accounting for the disproportionate distribution of infections in the localities with most of the co-infections observed in the Likoko locality.

Malnutrition prevalence, underweight and stunting varied significantly with sex and age with significantly higher occurrence of moderate and severe stunting in males and children 10–14 years. The prevalence of stunting is similar to that observed in SAC in Ghana [42], but higher than that observed in Northern Senegal [43] and in urban Cameroon [44]. The preponderance of linear growth retardation known as stunting in males has been well reported in Sub-Saharan Africa [45, 46]. Of note is the lower prevalence of *Plasmodium* infection and higher GMPD/ μL of blood in those malnourished or stunted with significant linear decline in infections with the severity of stunting. Similarly, the lowest prevalence of *S. haematobium* and co-infections and higher GMEC was observed in those with severe stunting. Despite the previous work by Olney et al. [47], which reported higher malaria parasite densities in children with lower height for age indices, Gari et al. [48] reported malaria as a risk factor for stunting although stunting was not associated with subsequent malaria illness. Stunting has been highlighted as a predictor of *S. haematobium* infection [42]. Whilst it is more likely that the severity of stunted malnutrition may have a negative influence on parasite density since both infections are transitory and severe stunting is chronic and prolonged nutritional inadequacies are required for it to manifest.

The hallmarks of *P. falciparum* infection include haematological alterations of which anaemia is the most common [49, 50] unlike in infections with *S. haematobium* and co-infections. The perpetual presence of anaemia in apparently healthy SAC negative for both infections with significantly higher occurrence in children 4–9 years old and those with co-infections necessitates caveat in attributing the causality of anaemia to these factors. Although the high prevalence of anaemia in those 4–9 years old could be linked to the high parasite densities of both *P. falciparum* and *S. haematobium* and microcytosis observed, the attributable risk of anaemia associated with these infections in the population remains low. Notwithstanding, findings from the multivariate analysis asserts a negative interaction between haemoglobin level with malaria parasites and *S. haematobium* as well as the association between haemoglobin and linear growth index.

While anaemia is one of the markers of morbidity associated with schistosomiasis even though it may be masked by anaemia resulting from malaria [51], the prevalence of anaemia in children co-infected with *P. falciparum* and *S. haematobium* was very high. This prevalence is similar with those in children in Ethiopia [52] with moderate anaemia being the most common form. Although the aetiology of anaemia is multifactorial, both infections may contribute to anaemia separately or through an interaction effect [51]. Nevertheless, the mechanism of malaria mediated anaemia has been well explained [53, 54] while anaemia by urogenital schistosomiasis may arise due to chronic blood loss as the egg penetrates the walls of the urinary tract, extra corporal loss of iron, autoimmune haemolysis and inflammation [55].

Findings from the study revealed that the mean Hct, WBC, lymphocyte and platelet counts, MCV, MCH, MCHC and RDW-CV were comparable amongst those infected with *P. falciparum* or *S. haematobium* or co-infection and those not infected. Infections with *P. falciparum* however, significantly lowered the Hb concentration and mean RBC counts. This effect is further bolstered by the significant association of *P. falciparum* with red cell indices including Hb, RBC and Hct in the multiple regression analysis. In line with findings by Kotepui et al. [50, 56], the negative association of *P. falciparum* infection with these red cell indices is not atypical and may be related to the enhanced destruction of infected and uninfected erythrocytes combined with decreased erythrocyte production leading to malaria related anaemia [57, 58].

Observations from the study highlighted age as a significant factor in most of the haematological parameters evaluated while sex was found to be associated with MCHC and RDW-CV. Since haematological parameters are interrelated with each other as well as with sex and age, of significance is the intricate positive interaction between the anthropometric malnutrition proxies of height-for-age and haematological parameters including haemoglobin, lymphocyte, MCH, MCHC and RDW-CV. As noted in previous studies [59, 60], the positive association suggests it is likely that the children suffer from chronic malnutrition (stunting) in addition to anaemia as well as systemic inflammation demonstrated by the relationship with lymphocytes and RDW-CV, an integrative measure of the pathological process. While a decrease in lymphocytes is implicated in infection with malaria parasite and anaemia, the biological function of different cell types including B lymphocytes has been reported to decrease during nutritional deficiencies [50, 61–63]. Nonetheless, the comparability of stunting to the population attributable risk of anaemia due to malaria and urogenital schistosomiasis demonstrates the ability to which stunting can exacerbate anaemia observed in the children.

Other haematological abnormalities of significance observed in the study population included thrombocytopenia, and microcytosis which were higher in SAC 4–9 years old. Furthermore, a higher occurrence of microcytosis was observed in males than females. Thrombocytopenia is commonly found in individuals living in areas endemic for *Schistosoma mansoni* while, microcytosis and thrombocytopenia is common in individuals with malaria [64–67]. Its association in individuals with *S. haematobium* is, however, uncertain. Although the context of interpretation is limited to the few cases of thrombocytopenia observed, findings from the study demonstrated no statistically significant association between platelet counts and parasite densities. It is worthy to note that children in the 4–9 years old age group had the highest parasite densities of both *Plasmodium* and *S. haematobium*. On the other hand, the high prevalence of microcytosis in the population may be attributed to the high prevalence of anaemia observed which is partly accounted for by the presence of the parasitic infections and chronic nutritional deficiencies.

The findings of the study should be viewed in the context of the limitations of the cross-sectional nature of the design which did not provide the changes in the burden and morbidities following control measures that could have been captured in a longitudinal study. Hence, there is a need for further investigations to ascertain the causal relationship between enhanced control interventions and changes in demographic as well as clinical characteristics and burden in this at-risk group to formulate more appropriate public health interventions.

## Conclusions

*Plasmodium falciparum* and *S. haematobium* infections are of public health concern while, malnutrition, microcytosis and thrombocytopenia are common, and anaemia is a severe public health problem in Muyuka, Cameroon. The interaction between haematological parameters with malaria parasites as well as linear growth index was negative even though the attributable risk of anaemia associated with these conditions remains low in the population. Other positive interactions between the linear growth index and haematological parameters indicate the occurrence of chronic malnutrition in addition to anaemia as well systemic inflammation. While findings provide contextual intervention targets to ensure the judicious use of the limited resources there is need for regular monitoring and proper treatment to improve the health of the underserved population.

## Supplementary Information

**Additional file 1.** Multiple linear regression analysis examining the influence of independent variables on some haematological parameters. Although not statistically significant UGS had a negative influence on Hb, Hct, WBC, RBC and lymphocyte counts, MCV, MCH and MCHC.

## Data Availability

All datasets generated and analysed in the current study are presented in the paper and supporting information file.

## Abbreviations

ACT: Artemisinin combination therapy
CI: Confidence interval
GMEC: Geometric mean egg count
GMPD: Geometric mean parasite density
Hct: Haematocrit
Hb: Haemoglobin
HA: Height-for-age
LLINs: Long-lasting insecticidal nets
MDA: Mass drug administration
MP: Malaria parasite
MCH: Mean cell haemoglobin
MCHC: Mean cell haemoglobin concentration
MCV: Mean cell volume
MLR: Multiple linear regression
NTD: Neglected Tropical Diseases
Plt: Platelet
RBC: Red blood cell
RDW-CV: Red cell distribution width-coefficient of variation
SAC: School-aged children
SD: Standard deviation
UGS: Urogenital schistosomiasis
WA: Weight-for-age
WH: Weight-for height
WBC: White blood cell
WHO: World Health Organisation
