# Supplementary material for: Confounding influences of malnutrition and Plasmodium falciparum and Schistosoma haematobium infections on haematological parameters in school children in Muyuka, Cameroon
Source: BMC Infect Dis. 2021 May 25;21:477. doi: 10.1186/s12879-021-06201-9 (PMC8152139; doi:10.1186/s12879-021-06201-9)
Supplement: Supplementary file 1 — Additional file 1. Multiple linear regression analysis examining the influence of independent variables on some haematological parameters. Although not statistically significant UGS had a negative influence on Hb, Hct, WBC, RBC and lymphocyte counts, MCV, MCH and MCHC. [file 12879_2021_6201_MOESM1_ESM.docx]

**Additional file 1.** **Multiple linear regression analysis examining the influence of independent variables on some haematological parameters**

| **Haematological variable** | **Independent variable** | **B** | **Standard error** | **95% CI** | **P value** | **Model summary** |
| --- | --- | --- | --- | --- | --- | --- |
| Hb in g/dL | Sex | -0.087 | 0.109 | -0.301-0.127 | 0.424 | R = 0.286 |
|  | Age | 0.169 | 0.026 | 0.118-0.220 | **<0.001** | R^2^ = 0.082 |
|  | HAZ | 0.117 | 0.040 | 0.038-0.196 | **0.004** | Adjusted R2 = 0.073 |
|  | MP Status | -0.379 | 0.152 | -0.677—0.082 | **0.013** | F = 8.904 |
|  | UGS status | -0.292 | 0.151 | -0.588-0.004 | 0.053 | P < 0.001 |
|  | Co-infection status | 0.306 | 0.275 | -0.235-0.847 | 0.267 |  |
| Hct in % | Sex | -0.510 | 0.308 | -1.115-0.094 | 0.098 | R = 0.282 |
|  | Age | 0.476 | 0.073 | 0.333-0.619 | **<0.001** | R^2^ = 0.080 |
|  | HAZ | 0.184 | 0.114 | -0.039-0.406 | 0.106 | Adjusted R^2^ = 0.070 |
|  | MP Status | -0.890 | 0.427 | -1.729—0.050 | **0.038** | F = 8.629 |
|  | UGS status | -0.641 | 0.426 | -1.477-0.195 | 0.133 | P < 0.001 |
|  | Co-infection status | 0.605 | 0.777 | -0.921-2.131 | 0.436 |  |
| WBC x 10 ^9^/L | Sex | 0.168 | 0.411 | -0.639-0.974 | 0.683 | R = 0.123 |
|  | Age | -0.207 | 0.098 | -0.399—0.015 | **0.034** | R^2^ = 0.015 |
|  | HAZ | 0.165 | 0.152 | -0.133-0.463 | 0.278 | Adjusted R^2^ = 0,005 |
|  | MP Status | -0.511 | 0.570 | -1.631-0.609 | 0.371 | F = 1.528 |
|  | UGS status | -0.567 | 0.568 | -1.682-0.549 | 0.319 | P = 0.166 |
|  | Co-infection status | 0.562 | 1.037 | -1.474-2.593 | 0.588 |  |
| RBC x 10^12^/L | Sex | -7194.0 | 41220.4 | -88148.4-73760.3 | 0.862 | R = 0.161 |
|  | Age | 31453.2 | 9785.1 | 12235.9-50670.4 | **0.001** | R^2^ = 0.026 |
|  | HAZ | 9208.9 | 15204.0 | -20650.8-39068.6 | 0.545 | Adjusted R^2^ = 0.016 |
|  | MP Status | -121790.0 | 57245.4 | -234216.5—9363.4 | **0.034** | F = 2.661 |
|  | UGS status | -69331.5 | 57002.2 | -181280.3-42617.2 | 0.224 | P < 0.015 |
|  | Co-infection status | 95391.4 | 104055.0 | -108966.2-299749.0 | 0.360 |  |
| Lymphocyte x 10^9^L | Sex | 0.267 | 0.223 | -0.171-0.705 | 0.231 | R = 157 |
|  | Age | -0.091 | 0.053 | -0.195-0.013 | 0.085 | R^2^ = 0.025 |
|  | HAZ | 0.200 | 0.082 | 0.038-0.361 | **0.015** | Adjusted R^2^ = 0.015 |
|  | MP Status | -0.135 | 0.310 | -0.743-0.473 | 0.664 | F = 2.535 |
|  | UGS status | -0.400 | 0.308 | -1.005-0.205 | 0.195 | P < 0.020 |
|  | Co-infection status | 0.186 | 0.563 | -0.919-1.291 | 0.741 |  |
| MCV in fL | Sex | -1.042 | 0.501 | -2.025—0.058 | 0.038 | R = 0.213 |
|  | Age | 0.566 | 0.119 | 0.332-0.799 | **<0.001** | R^2^ = 0.046 |
|  | HAZ | 0.281 | 0.185 | -o.82-0.644 | 0.129 | Adjusted R^2^ = 0.036 |
|  | MP Status | -0.079 | 0.696 | -1.445-1.287 | 0.910 | F = 4.754 |
|  | UGS status | -0.397 | 0.693 | -1.758-0.963 | 0.566 | P < 0.001 |
|  | Co-infection status | -0.143 | 1.265 | -2.627-2.340 | 0.910 |  |
| MCH in pg | Sex | -0.153 | 0.180 | -0.506-0.200 | 0.394 | R = 0.226 |
|  | Age | 0.209 | 0.043 | 0.125-0.293 | **<0.001** | R^2^ = 0.051 |
|  | HAZ | 0.230 | 0.066 | 0.099-0.360 | **0.001** | Adjusted R^2^ = 0.042 |
|  | MP Status | -0.143 | 0.250 | -0.633-0.347 | 0.567 | F = 5.369 |
|  | UGS status | -0.287 | 0.249 | -0.775-0.201 | 0.249 | P < 0.001 |
|  | Co-infection status | 0.169 | 0.454 | -0.722-1.060 | 0.710 |  |
| MCHC in g/L | Sex | 0.333 | 0.166 | 0.006-0.660 | **0.046** | R = 0.141 |
|  | Age | 0.011 | 0.039 | -0.067-0.088 | 0.787 | R^2^ = 0.020 |
|  | HAZ | 0.175 | 0.061 | 0.055-0.296 | **0.004** | Adjusted R^2^ = 0.010 |
|  | MP Status | -0.190 | 0.231 | -0.643-0.264 | 0.412 | F = 2.032 |
|  | UGS status | -0.225 | 0.230 | -0.676-0.227 | 0.329 | P = 0.060 |
|  | Co-infection status | 0.333 | 0.420 | -0.492-1.157 | 0.429 |  |
| RDW-CV% | Sex | 0.372 | 0.109 | 0.158-0.586 | **0.001** | R = 0.233 |
|  | Age | -0.101 | 0.026 | -0.152—0.050 | **<0.001** | R^2^ = 0.54 |
|  | HAZ | -0.091 | 0.040 | -0.170—0.013 | **0.023** | Adjusted R^2^ = 0.045 |
|  | MP Status | 0.077 | 0.151 | -022-0.374 | 0.612 | F = 5.718 |
|  | UGS status | 0.292 | 0.151 | -0.004-0.588 | 0.053 | P < 0.001 |
|  | Co-infection status | -0.330 | 0.275 | -0.870-0.210 | 0.231 |  |

P values in bold are statistically significant
